# Anti-Inflammatory Effects of Secondary Metabolites of Marine *Pseudomonas* sp. in Human Neutrophils Are through Inhibiting P38 MAPK, JNK, and Calcium Pathways

**DOI:** 10.1371/journal.pone.0114761

**Published:** 2014-12-04

**Authors:** Shun-Chin Yang, Ping-Jyun Sung, Chwan-Fwu Lin, Jimmy Kuo, Chun-Yu Chen, Tsong-Long Hwang

**Affiliations:** 1 Graduate Institute of Biomedical Sciences, College of Medicine, Chang Gung University, Taoyuan, Taiwan; 2 Department of Anesthesiology, Taipei Veterans General Hospital, Taipei, Taiwan; 3 Department of Life Science and Graduate Institute of Biotechnology, Graduate Institute of Marine Biotechnology, National Dong Hwa University, Pingtung, Taiwan; 4 Department of Cosmetic Science, and Research Center for Industry of Human Ecology, Chang Gung University of Science and Technology, Taoyuan, Taiwan; 5 National Museum of Marine Biology & Aquarium, Pingtung, Taiwan; 6 Graduate Institute of Clinical Medical Sciences, College of Medicine, Chang Gung University, Taoyuan, Taiwan; 7 Department of Anesthesiology, Chang Gung Memorial Hospital, Taoyuan, Taiwan; 8 Graduate Institute of Natural Products, School of Traditional Medicine, College of Medicine, and Chinese Herbal Medicine Research Team, Healthy Aging Research Center, Chang Gung University, Taoyuan, Taiwan; Institute of Biochemistry and Biotechnology, Taiwan

## Abstract

Activated neutrophils play a significant role in the pathogenesis of many inflammatory diseases. The metabolites of marine microorganisms are increasingly employed as sources for developing new drugs; however, very few marine drugs have been studied in human neutrophils. Herein, we showed that secondary metabolites of marine *Pseudomonas* sp. (N11) significantly inhibited superoxide anion generation and elastase release in formyl-L-methionyl-L-leucyl-L-phenylalanine (FMLP)-activated human neutrophils, with IC_50_ values of 0.67±0.38 µg/ml and 0.84±0.12 µg/ml, respectively. In cell-free systems, neither superoxide anion-scavenging effect nor inhibition of elastase activity was associated with the suppressive effects of N11. N11 inhibited the phosphorylation of p38 MAP kinase and JNK, but not Erk and Akt, in FMLP-induced human neutrophils. Also, N11 dose-dependently attenuated the transient elevation of intracellular calcium concentration in activated neutrophils. In contrast, N11 failed to alter phorbol myristate acetate-induced superoxide anion generation, and the inhibitory effects of N11 were not reversed by protein kinase A inhibitor. In conclusion, the anti-inflammatory effects of N11 on superoxide anion generation and elastase release in activated human neutrophils are through inhibiting p38 MAP kinase, JNK, and calcium pathways. Our results suggest that N11 has the potential to be developed to treat neutrophil-mediated inflammatory diseases.

## Introduction

Neutrophils are major cells that induce innate immune responses and they also act as components of cellular inflammatory reactions [1]. Neutrophils are recruited to inflammatory areas in response to stimuli, and subsequently kill the invasion pathogens through respiratory burst and degranulation [2]. However, growing evidence has suggested that overwhelming activation of neutrophils is harmful to human health. Human neutrophils play a critical role not only in infective inflammation but also in sterile inflammation [3], [4], [5]. Recently emerging evidence has suggested that inhibition of activation of human neutrophils is a viable therapeutic strategy for the treatment of organ injuries and inflammatory diseases [6], [7].

Mitogen-activated protein (MAP) kinases, which consist of Erk, p38 kinase, JNK and big MAP kinase-1, are closely related to regulation of inflammatory process, such as inflammatory cytokines release and reactive oxygen species (ROS) production [8]. Recent research has demonstrated that MAP kinases are potential therapeutic targets for the treatment of inflammatory diseases [9]. Notably, the inhibitors of p38 MAP kinase are able to prevent the progression of collagen-induced arthritis, inflammatory bowel disease, and chronic obstructive pulmonary disease [10], [11], [12]. However, the undesired side effects of these inhibitors limited their clinical development and other potent compounds remained to be explored.

Extracellular products from terrestrial and marine microorganisms have yielded an increasing source of new compounds for use in drug development [13]. Microorganisms produce numerous extracellular metabolites that can affect tumor cell viability, bacteria growth, and immune cell functions. Recently, our and other studies have shown various biologic effects of secondary metabolites from marine microorganisms, including anti-bacterial, anti-tumor and anti-inflammatory effects [14], [15], [16], [17]. However, studies related to the pharmacologic mechanisms on anti-inflammatory effects of these secondary metabolites in human neutrophils remained ambiguous.

In this study, we show for the first time that anti-inflammatory effect of the bioactive metabolites of marine *Pseudomonas* sp. (N11) on respiratory burst and degranulation in activated human neutrophils. The pharmacologic mechanisms of N11 in activated human neutrophils were further investigated. The signal transduction cascade responsible for regulating neutrophil activation is very complex and remains to be completely defined. *N*-formyl peptides, which originated from either bacteria or mitochondria, have been regarded as strong chemoattractants for neutrophils [18], [19], [20]. Formyl-L-methionyl-L-leucyl-L-phenylalanine (FMLP) is one of the *N*-formyl peptides and has been well recognized for studying the pathologic effects of neutrophils. FMLP induced intracellular calcium mobilization and phosphorylation of MAP kinases and Akt protein [21], [22]. Our results demonstrated that anti-inflammatory effects of N11 are at least partially attributed to the inhibition of intracellular calcium mobilization and phosphorylation of p38 MAP kinase and JNK.

## Materials and Methods

### Reagents

Fluo-3/AM was obtained from Molecular Probes (Eugene, OR, USA). Methoxysuccinyl-ala-ala-pro-val-nitroanilide and *N*-[2-(p-bromocinnamylamino)ethyl]-5-isoquinolinesulfonamide (H89) were purchased from Calbiochem (La Jolla, CA, USA). Antibodies against phospho-p38, phospho-Erk, Erk, phospho-JNK, JNK, phospho-Akt (ser-473), and Akt (pan) were purchased from Cell Signaling (Beverly, MA, USA). The antibody against p38 MAP kinase was obtained from Santa Cruz Biotechnology (Santa Cruz, CA, USA). 2-(4-Iodophenyl)-3-(4-nitrophenyl)-5-(2,4-disulfophenyl)-2 H-tetrazolium monosodium salt (WST-1) was obtained from Dojindo Laboratories (Kumamoto, Japan). All other pharmacologic agents were purchased from Sigma-Aldrich (St. Louis, MO, USA).

### Isolation of human neutrophils

The Chang Gung Medical Foundation Institutional Review Board (IRB number: 99–3848B) specifically approved this study. Each volunteer provided his or her written informed consent. Venous blood was collected from healthy volunteers who had not taken any drugs within at least 2 weeks. Neutrophils were isolated from the peripheral blood using the standard dextran sedimentation method prior to centrifugation in a Ficoll Hypaque gradient and the hypotonic lysis of the erythrocytes. Purified neutrophils contained >98% viable cells, which determined by using trypan blue exclusion method. Neutrophils were stored at 4°C before use and were suspended in Hank’s balanced salt solution without calcium.

### Bacterial strains, cultivation condition, and preparation

The samples were prepared according to the methods used in a previous study [15]. In brief, the bacterial strain SLI-02–04 was isolated from marine sediment collected at the Siao Lanyu Isle of Taiwan (21°57′34.58″N, 121°37′11.88″E). The strain was identified as *Pseudomonas* sp. after conducting a 16S rDNA analysis. The 16S rDNA sequence of SLI-02–04 was deposited in the National Center for Biotechnology Information Genbank under accession number KC865054. It was maintained on M1 agar (10 g of starch, 4 g of yeast extract, 2 g of peptone, 0.5 L of seawater, 0.5 L of dH_2_O, and 15 g of agar) at 25°C in Petri dishes. *Pseudomonas* sp. was aerobically cultivated in 2 L-ml flasks containing 1000 ml of M1 medium and 50% seawater. The flasks were incubated at 25°C on a rotatory shaker at 150 rpm. After 5 d of incubation, the fermented broths were extracted twice using ethyl acetate. The solvent extracts were combined and evaporated to dryness in a vacuum. The extracts (N11) thus obtained were weighed and stored at −20°C prior to use in bioactivity assays.

### Fingerprint chromatogram of N11

The high performance liquid chromatography (HPLC) fingerprint of N11 was conducted on a Hitachi HPLC system (L-2000 series, Tokyo, Japan). The concentration of N11 was 4 mg/ml. The separation was performed using a Cosmosil 5C18-AR-II column (5 µm, 25 cm×4.6 mm I.D.) at an elution flow rate of 0.8 ml/min and with a mix solvent of A-B (A = H_2_O, B = CH_3_CN), which was varied as follows: 0–10 min, 98% A, 2% B; 10–15 min, 98–0% A, 2–100% B; 15–30 min, 0% A, 100% B. The injection volume was 10 µl, and the UV detection wavelength was set at 220 nm. Ultrapure water and acetonitrile elution solvent were used as mobile phases in a series of experiments. Identification of N11 was dependent on retention time and UV spectra in comparison with the standard.

### Measurement of superoxide anion generation and elastase release

The superoxide anion generation in activated human neutrophils was measured using the reduction of ferricytochrome *c.* In addition, Methoxysuccinyl-Ala-Ala-Pro-Val-p-nitroanilide was used as the elastase substrate to detect elastase release. Neutrophils were incubated with ferricytochrome *c* or elastase substrates at 37°C, and then treated with N11 for 5 min. FMLP (30 nM) with cytochalasin B (0.5 or 1 µg/ml) or phorbol myristate acetate (PMA, 5 nM) was used to stimulate neutrophils. The change in absorbance was continually monitored at 550 nm or 405 nm using a spectrophotometer (U-3010, Hitachi, Tokyo, Japan) [23], [24].

Neutrophils were incubated with FMLP (100 nM) for 15 min and supernatants were collected for extracellular elastase activity assay. The supernatants were incubated with DMSO (as the control group) or testing drugs for 2 min and reacted with elastase substrate for 10 min. The change in absorbance was continually monitored at 405 nm by spectrophotometry.

Hydroethidine (HE) was employed to detect intracellular ROS production. Neutrophils were labeled with HE (10 µM) for 15 min at 37°C. HE-labeled neutrophils were treated with N11 for 5 min before adding FMLP (30 nM). The fluorescence intensity was assayed using flow cytometry.

### Superoxide anion and DPPH scavenging assay

The superoxide anion-scavenging effect of N11 was examined in a cell-free xanthine/xanthine oxidase system. The assay buffer contained 50 mM Tris (pH 7.4), 0.3 mM WST-1, and 0.02 U/ml xanthine oxidase. WST-1 was reduced by superoxide anion after adding 0.1 mM xanthine to the assay buffer at 30°C. The absorbance was measured at 450 nm. In addition, the antioxidant ability was determined by DPPH assay. An ethanol solution of DPPH (100 µM) was incubated with N11 at 30°C. The absorbance was subsequently measured at 517 nm.

### Evaluation of lactate dehydrogenase (LDH) release

LDH release was determined using a commercially available method (Promega, Madison, WI, USA). The calculation was made according to the LDH activity in the presence or absence of N11, which was expressed as a percentage of the total LDH activity. The total LDH activity was determined by lysing the neutrophils with 0.1% Triton X-100 at 37°C.

### Measurement of [Ca^2+^]_i_

Human neutrophils were labeled with Fluo-3/AM (2 µM) for 30 min at 37°C. The cytoplasmic calcium level was measured using a thermostat in a quartz cuvette while undergoing continuous stirring with a Hitachi F-4500 spectrofluorometer. The excitation wavelength was 488 nm and the emission wavelength was 520 nm. After the cells were treated with N11 for 5 min, FMLP (30 nM) was added to induce the peak [Ca^2+^]_i_. The [Ca^2+^]_i_ was then calculated according to the fluorescence intensity, as follows: [Ca^2+^]_i_ = Kd×[(F - F_min_)/(F_max_ - F)]; where F is the observed fluorescence intensity; F_max_ and F_min_ were obtained by adding 0.05% Triton X-100 and 20 mM EGTA, respectively, to the neutrophils; and Kd was 400 nM.

### Immunoblotting analysis

After incubated with N11 for 5 min, neutrophils were stimulated with FMLP (30 nM) for 30 sec, and then mixed with sample buffer for 15 min at 100°C to stop the reaction. After centrifugation at 14, 000×g for 20 min at 4°C, whole-cell lysates were produced. To separate the proteins, sodium dodecyl sulfate-polyacrylamide gel electrophoresis with 12% polyacrylamide gels were used. The samples were then blotted onto nitrocellulose membranes. Immunoblotting was performed using the indicated primary antibodies and horseradish peroxidase-conjugated secondary anti-rabbit antibodies (Cell Signaling Technology, Beverly, MA, USA). The immunoreactive bands were visualized using an enhanced chemiluminescence system (Amersham Biosciences, Piscataway Corp., NJ, USA). The intensities of these bands were analyzed using UVP Biospectrum (UVP, LLC Upland, CA, USA).

### Statistical analysis

All results are expressed as means ± SEM. A one-way ANOVA analysis followed by Bonferroni’s *post hoc* test was used for all experiments. SigmaPlot (Systat Software, San Jose, CA) was used for all analyses. A value of *p*<0.05 was considered significant.

## Results

### Fingerprint chromatogram of N11

**Figure 1:**
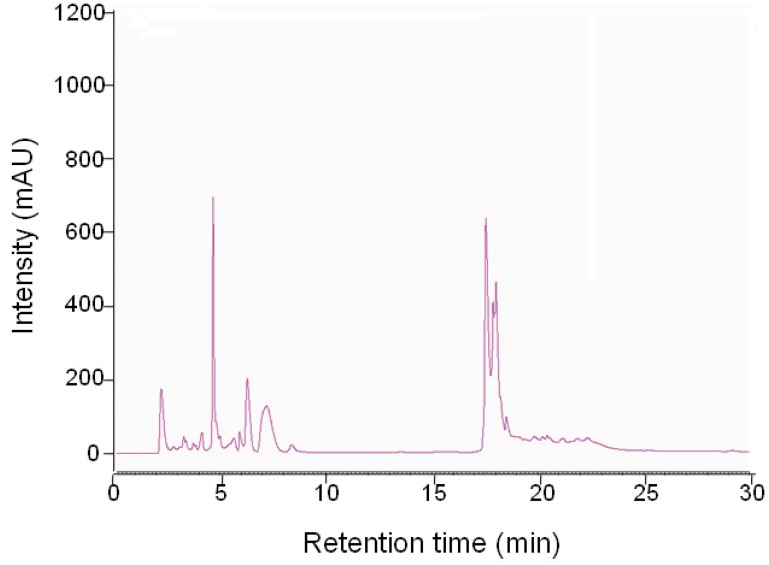
Chromatography analysis of N11. *Pseudomonas* sp. was aerobically cultivated in culture medium with 50% seawater at 25°C on a rotatory shaker at 150 rpm for 5 d. The metabolites were extracted using ethyl acetate and evaporated to dryness in a vacuum to obtain N11. The concentration of N11 for HPLC analysis was 4 mg/ml. The injection volume was 10 µl, and the UV detection wavelength was set at 220 nm.

The fingerprint chromatogram of N11 was obtained by HPLC for quality control of the ethyl acetate extraction of secondary metabolites of marine *Pseudomonas* sp. In a full-scan assay, the detector wavelength at 220 nm showed superior separation compared with other wavelengths. The fingerprint chromatogram displayed a group of peaks at polar fraction (2.2–8.6 min RT, elution solvent: 2% CH_3_CN) and another group of peaks at non-polar fraction (17.4–23.0 min RT, elution solvent: 100% CH_3_CN) (Figure 1).

### N11 reduces superoxide anion generation in FMLP-activated human neutrophils

**Figure 2:**
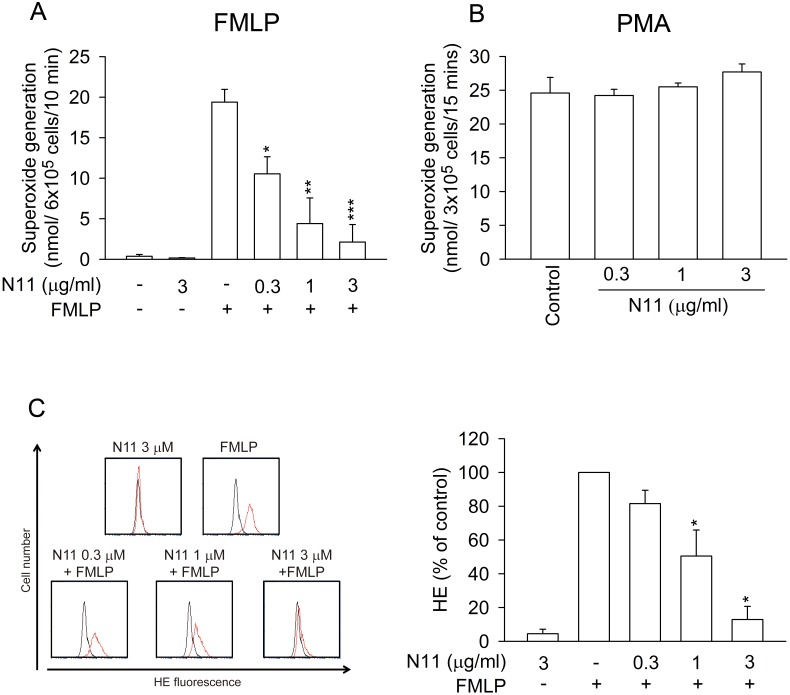
N11 suppresses superoxide anion generation and ROS formation in FMLP-activated human neutrophils. Neutrophils were incubated with DMSO (control) or N11 (0.3, 1, and 3 µg/ml) and then stimulated with (A) FMLP (30 nM) or (B) PMA (5 nM). Superoxide anion generation was measured by spectrophotometry. (C) Neutrophils labeled with HE were incubated with DMSO (control) or N11 (0.3, 1, and 3 µg/ml) and monitored by flow cytometry under resting and stimulating conditions. The black line denotes the basal group comprising cells treated with DMSO without FMLP stimulation. The red line denotes the experimental groups. All data shown are means ± SEM. (n = 5 for A, n = 3 for B, n = 4 for C). * *p*<0.05, ** *p*<0.01, *** *p*<0.001 versus the control group.

It is well known that excess generation of ROS can trigger tissue injury [25], [26], [27]. Spectrophotometry and flow cytometry were employed to determine whether N11 altered the generation of superoxide anion and ROS in activated human neutrophils. N11 significantly inhibited superoxide anion generation and ROS formation in FMLP-activated neutrophils, with IC_50_ values of 0.67±0.38 and 1.23±0.35 µg/ml, respectively (Figure 2A and 2C). N11 did not alter basal superoxide anion generation in resting conditions. In contrast, N11 failed to affect superoxide anion generation in PMA, a protein kinase C (PKC) activator, stimulated human neutrophils (Figure 2B), suggesting that the inhibitory effect of N11 on superoxide anion generation is mediated by a PKC-independent pathway.

### N11 fails to suppress ROS generation in cell-free systems

**Figure 3:**
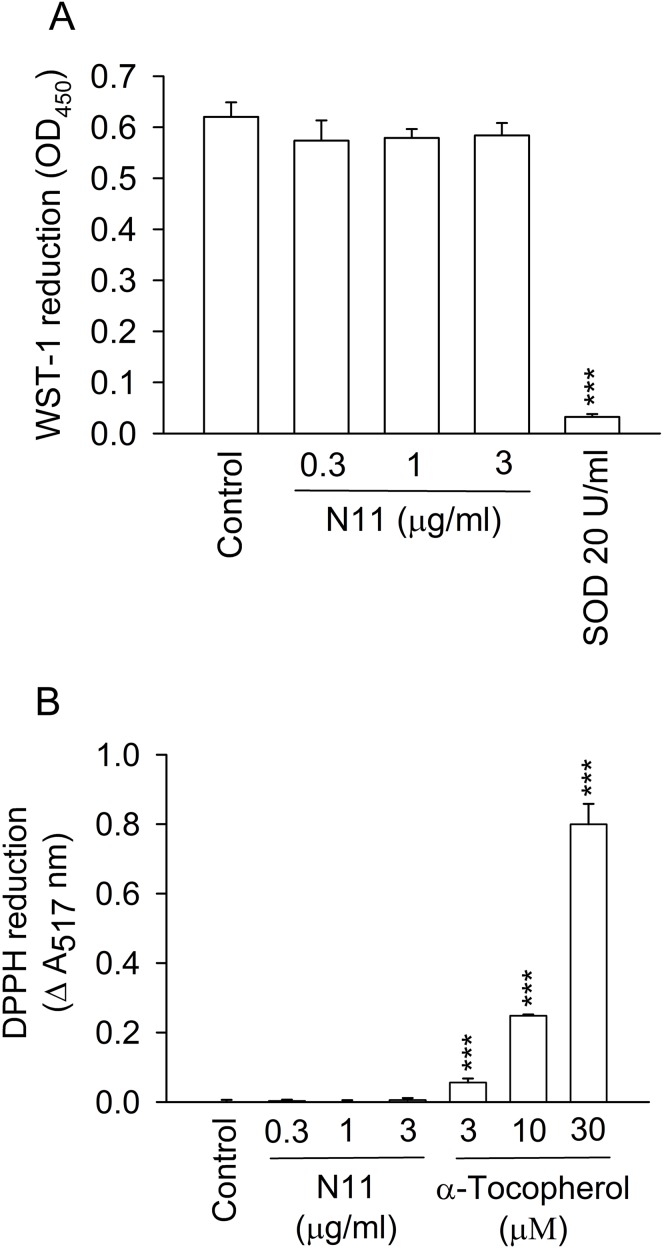
N11 does not have superoxide anion-scavenging ability and antioxidant effect in cell-free systems. (A) Reduction of WST-1 by superoxide anion in xanthine/xanthine oxidase assay and (B) reduction of DPPH radical in the presence of N11, SOD, or α-tocopherol, were measured by spectrophotometry. All data shown are means ± SEM. (n = 3 for A, n = 4 for B). *** *p*<0.001 versus the control group.

The cell-free xanthine/xanthine oxidase system and DPPH assay were employed to determine whether N11 exhibited superoxide anion-scavenging or antioxidant properties. N11 at dose up to 3 µg/ml did not affect superoxide anion generation and DPPH reduction in cell-free systems. Superoxide dismutase (SOD) and α-tocopherol were used as positive controls, respectively (Figure 3A and 3B). In addition, N11 did not induce release of LDH, indicating that N11 exhibited no membrane damage and cytotoxicity (data not shown).

### N11 inhibits elastase release in activated human neutrophils

**Figure 4:**
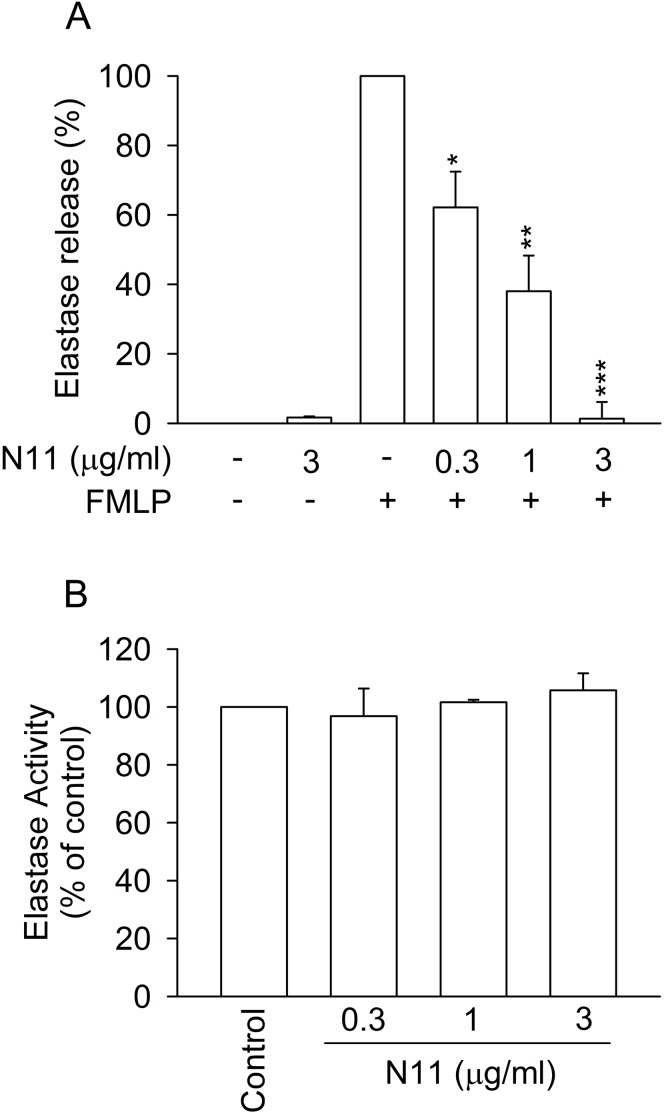
N11 inhibits elastase release in FMLP-activated human neutrophils. (A) Neutrophils were incubated with DMSO (control) or N11 (0.3, 1, and 3 µg/ml) and then stimulated with FMLP (30 nM). Elastase release was measured by spectrophotometry. (B) Elastase supernatant was incubated with DMSO (control) or N11 before the addition of substrate. All data shown are means ± SEM. (n = 3 for A, n = 5 for B). * *p*<0.05, ** *p*<0.01, *** *p*<0.001 versus the control group.

The degranulation of neutrophil granules, such as elastase release, is another crucial immune response in activated neutrophils [28], [29]. Therefore, diminishing the release or activity of neutrophil elastase is crucial for treating inflammatory disorders. N11 significantly inhibited elastase release in FMLP-activated neutrophils, with an IC_50_ value of 0.84±0.12 µg/ml (Figure 4A). By contrast, N11 did not directly alter the activity of elastase in a cell-free assay (Figure 4B). The results suggested that the inhibitory effect of N11 on elastase release is through the modulation of intracellular signaling pathways.

### N11 inhibits FMLP-induced transient elevation of intracellular calcium concentration ([Ca^2+^]_i_)

**Figure 5:**
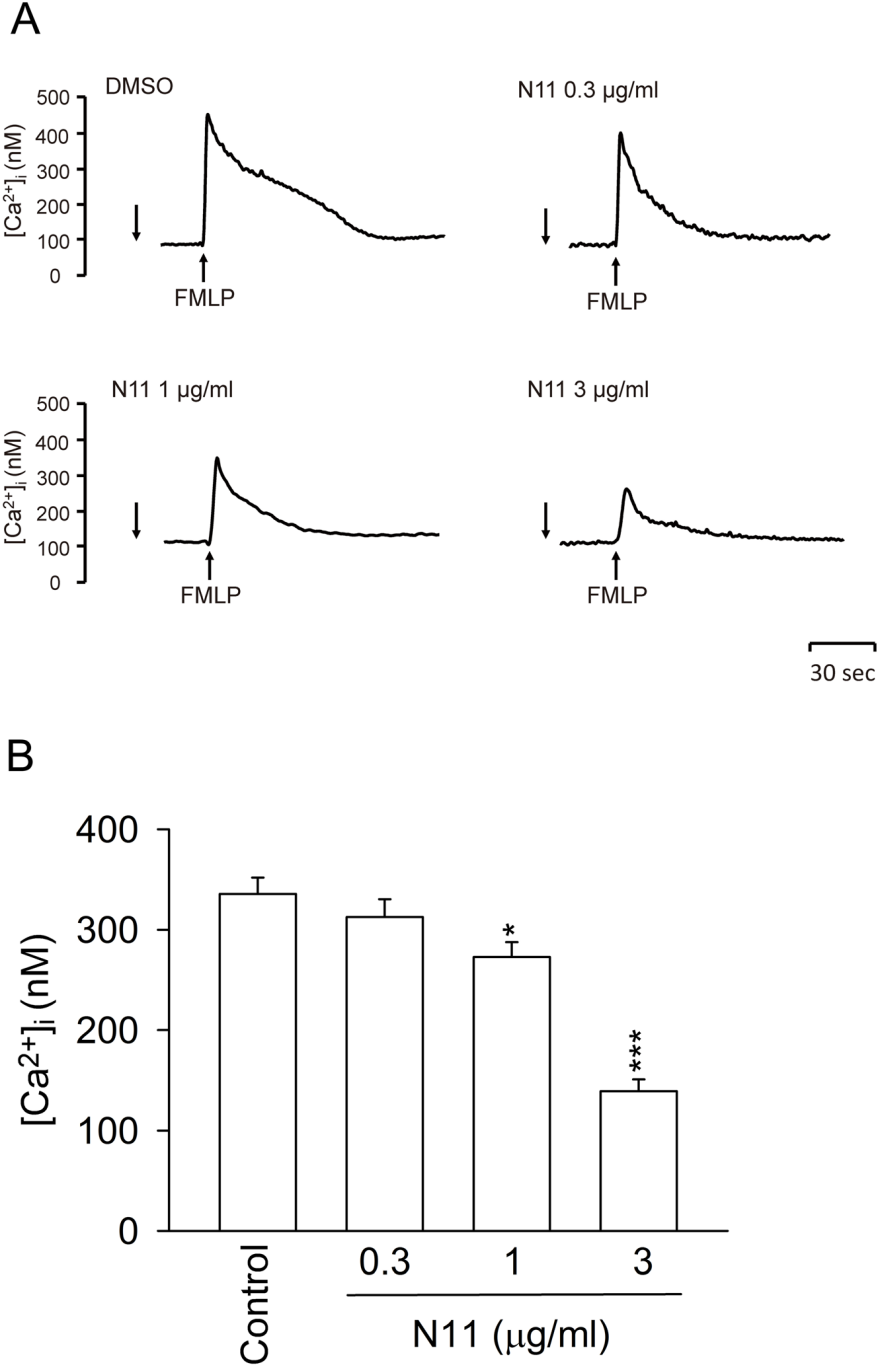
N11 down-regulates calcium mobilization in FMLP-activated human neutrophils. (A) Fluo-3/AM loaded neutrophils were incubated with DMSO (control) or N11 and then activated with FMLP (30 nM). Mobilization of calcium was determined in real time in a spectrofluorometer. Representative traces from one of six experiments are shown. (B) Peak [Ca^2+^]_i_ induced by FMLP is expressed as means ± SEM. (n = 6). * *p*<0.05, *** *p*<0.001 versus the control group.

The present data showed that N11 possesses anti-inflammatory effects, including suppressing superoxide anion generation, ROS formation, and elastase release, in FMLP-activated human neutrophils. Calcium signals play crucial roles in the regulation of superoxide anion generation and elastase release in activated human neutrophils [30]. FMLP induced the transient elevation of [Ca^2+^]_i_ in human neutrophils. Notably, peak [Ca^2+^]_i_ was dose-dependently inhibited by N11 in FMLP-activated neutrophils (Figure 5). These data suggested that the mechanism behind the anti-inflammatory effects of N11 in human neutrophils may be related to calcium signaling pathways.

### Protein kinase A (PKA) is not involved in the inhibitory effects of N11

**Figure 6:**
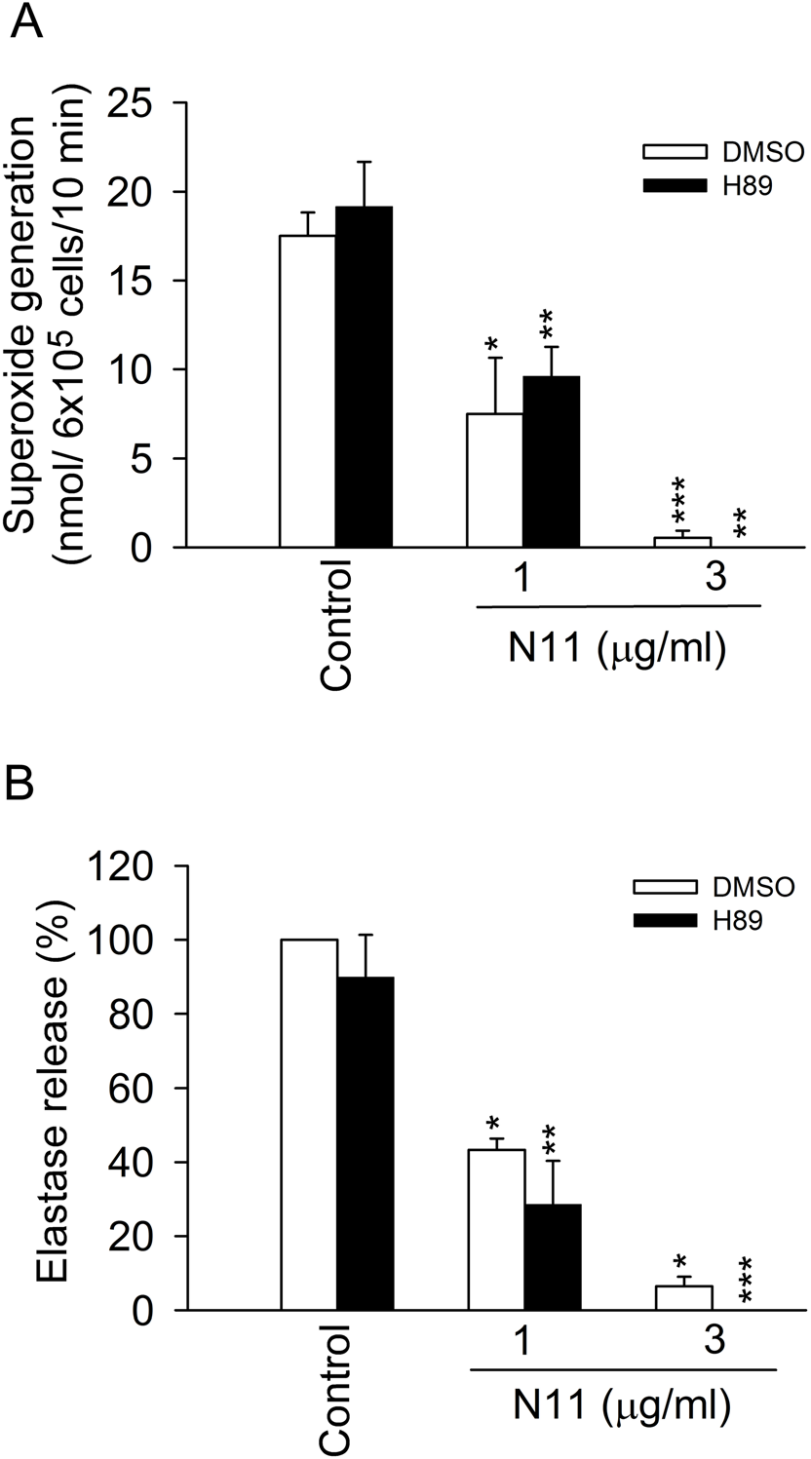
PKA is not involved in N11-caused inhibition. (A) Superoxide anion generation and (B) elastase release in FMLP-activated human neutrophils were examined. H89 (PKA inhibitor, 3 µM) was preincubated before the addition of N11. All data shown are means ± SEM. (n = 4). * *p*<0.05, ** *p*<0.01, *** *p*<0.001 versus the corresponding control group.

Our and other studies have demonstrated that the activation of intracellular cAMP/PKA pathways has a negative modulatory effect on neutrophil functions [23], [31], [32]. Figure 6A and 6B show that H89, a PKA inhibitor, did not reverse the inhibitory effects of N11 on superoxide anion generation and elastase release. Collectively, the anti-inflammatory effects of N11 in activated neutrophils were not associated with PKA pathway.

### N11 attenuates phosphorylation of p38 MAP kinase and JNK but not Erk and Akt in FMLP-activated human neutrophils

**Figure 7:**
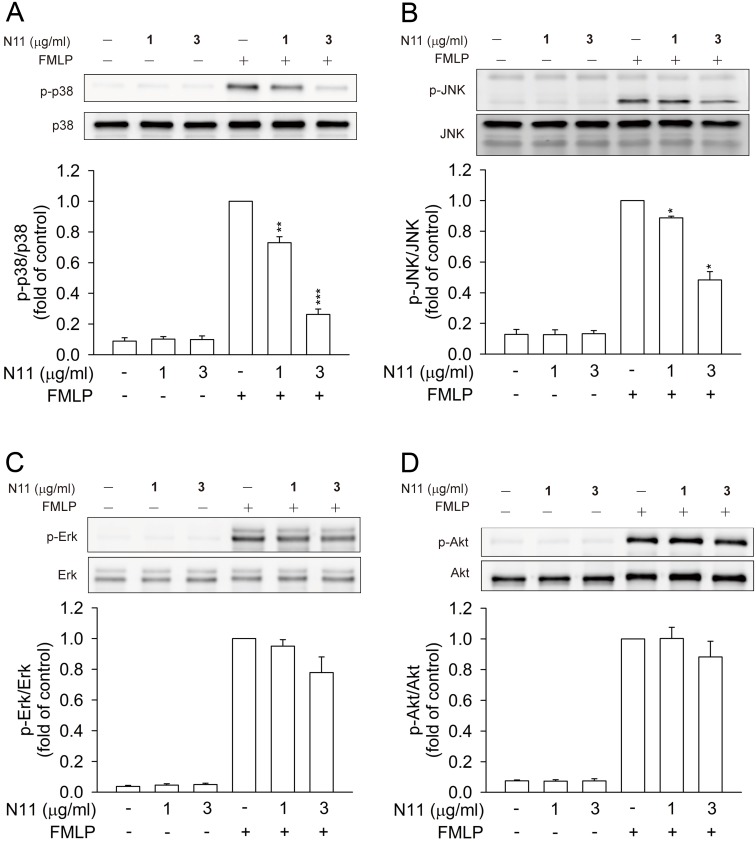
N11 inhibits phosphorylation of p38 and JNK, but not Erk and Akt, in FMLP-activated human neutrophils. Neutrophils were treated with N11 (1 and 3 µg/ml) and then activated with FMLP (30 nM). Phosphorylation of MAP kinases and Akt were analyzed by immunoblotting analysis. Densitometric analysis of all samples was normalized to the total protein. All data shown are means ± SEM. (n = 4). * *p*<0.05, ** *p*<0.01, *** *p*<0.001 versus the control group.

In addition to calcium and PKA signals, MAP kinases and Akt pathways play significant roles in modulating neutrophil functions; furthermore, the inhibition of these proteins has been shown to diminish organ damage [33], [34]. As shown in Figure 7, MAP kinases and Akt protein were phosphorylated in FMLP-activated neutrophils. Significantly, N11 attenuated the FMLP-induced phosphorylation of p38 MAP kinase and JNK in human neutrophils (Figure 7A and 7B). In contrast, N11 failed to alter the phosphorylation of Erk and Akt (Figure 7C and 7D). These results demonstrated that the inhibitory effects of N11 are mediated by the decrease of phosphorylated p38 MAP kinase and JNK levels.

## Discussion

There is growing evidence that overproduction of neutrophil inflammatory responses can be harmful to human health [35], [36], [37]. In this study, human neutrophils were used to determine whether N11, the extraction of secondary metabolites of marine *Pseudomonas* sp., could attenuate inflammatory responses. Our results demonstrated that N11 significantly inhibited superoxide anion generation and elastase release in FMLP-activated human neutrophils in dose-dependent manners. The anti-inflammatory effects of N11 are mediated by the inhibition of intracellular calcium mobilization and phosphorylation of p38 MAP kinase and JNK in FMLP-induced human neutrophils.

ROS generated from activated human neutrophils not only eliminate invasion pathogens but also trigger inflammatory processes. The oxidative stress caused by activated human neutrophils plays a crucial role in the pathogenesis of inflammatory diseases, including sepsis, ischemia/reperfusion injury, and autoimmune diseases [2], [38], [39]. Accordingly, ROS are potential therapeutic targets to treat inflammatory disorders. Our and other studies have showed that the bioactive compounds from marine sponge, ascidian, coral, and alga exert anti-inflammatory abilities by suppressing ROS release from activated neutrophils [40], [41], [42], [43]. Moreover, some compounds from marine *Pseudomonas* sp., have also been shown to exert antioxidant and anti-inflammatory effects [44], [45]. Our results showed that N11 exerted anti-inflammatory effects by significantly inhibiting superoxide anion generation and ROS formation in FMLP-activated human neutrophils. In contrast to previous findings, however, our results demonstrated that N11 did not have superoxide-scavenging effect and antioxidant ability. Therefore, we suggest that the suppressive effects of N11 are mediated by modulation of intracellular signaling pathways.

In addition to oxidative stress, substantial evidences have demonstrated that acute and chronic inflammatory disorders, such as chronic obstructive pulmonary disease, inflammatory bowel disease, and cardiovascular disease, are caused by elevated level of neutrophil elastase [29], [46], [47], [48]. A study on bioactive compounds from marine cyanobacteria, *Lyngbya* spp. has suggested that several marine compounds may have therapeutic potency by selective inhibition of elastase activity [49]. The present study showed that N11 significantly inhibited FMLP-induced elastase release, but not elastase activity, in human neutrophils. Taken together, these data support our hypothesis that N11 displays anti-inflammatory abilities.

FMLP is a well-known chemoattractant to activate formyl peptide receptor 1 (FPR1), a G-protein coupled receptor [50], [51]. FPR1 is recognized by *N*-formyl peptides, which originated from either bacteria or mitochondria, to trigger infective and sterile inflammation [52], [53]. In addition, PMA directly triggers PKC to induce NADPH oxidase activation and ROS generation in human neutrophils [54]. Interestingly, our results showed that N11 specifically inhibited respiratory burst in FMLP-stimulated neutrophils, but not in PMA-treated cells. These results suggest that N11 exerts its anti-inflammatory effects upstream of PKC. In FMLP-activated human neutrophils, the level of intracellular inositol 1,4,5-triphosphate is elevated to induce transient calcium release from endoplasmic reticulum. In this study, significantly inhibition of calcium mobilization in FMLP-activated neutrophils was observed in the presence of N11. On the other hand, phosphatidylinositol-3-kinase (PI3K)/Akt and MAP kinases are known to be responsible for various neutrophil responses [55]. Activation of PI3K/Akt pathway in stimulated neutrophils plays a significant role on superoxide anion generation and elastase release [56]. Phosphorylation of p38 MAP kinase and JNK also contribute towards neutrophil activation [57], [58]. Significantly, our results showed that the anti-inflammatory effects of N11 are through attenuating the phosphorylation of p38 MAP kinase and JNK, but not Erk and Akt, in FMLP-stimulated human neutrophils. Moreover, the attenuation of intracellular calcium concentration and phosphorylation of p38 and JNK was slightly in the presence of low dose of N11, indicating that the suppressive effects of N11 on human neutrophil activations were through inhibition of multiple mechanisms.

## Conclusions

In conclusion, the present study demonstrates that N11, the secondary metabolites of marine *Pseudomonas* sp., significantly inhibits human neutrophil respiratory burst and degranulation. The anti-inflammatory effects are mediated by multiple mechanisms, including inhibition of intracellular calcium level and reduction in phosphorylation of p38 MAP kinase and JNK. Elucidating the structures of bioactive compounds derived from the secondary metabolites of marine *Pseudomonas* sp. remains an ongoing process. In view of the central role of activated human neutrophils in various inflammatory diseases, identifying bioactive metabolites and compounds from marine bacteria may provide potential medical treatment options.
